# Giant cell tumor of bone with secondary aneurysmal bone cyst

**DOI:** 10.4103/0973-6042.42582

**Published:** 2008

**Authors:** Chetan Anchan

**Affiliations:** Bombay Hospital and Medical Research Center, Room No.207, 2nd Floor, New Wing, 12 New Marine Lines, Mumbai - 400 020, India

The importance of diagnosing giant cell tumor (GCT) of bone correctly is that despite its aggressive clinical behavior it is a benign tumor. In most cases GCT of bone is treated with intralesional surgery which is thorough curettage along with use of adjuvants and appropriate reconstruction. In orthopedic oncology, to come to a diagnosis of any bone tumor, the clinical presentation and behavior of the tumor, its radiological appearance, and its histopathology should be in agreement. Any discrepancy should immediately raise suspicion, and warrants a review of the findings jointly by the clinician, the radiologist, and the pathologist. Though its name may imply that the characteristic large osteoclastic giant cells of a GCT of bone are responsible for the proliferative capacity of the tumor, there is evidence that the stromal-like cells which are the major component of the mononuclear cell population represent the true neoplastic component of the tumor.[1]

It is noteworthy that the authors have pointed out that it is important to rule out giant cell-rich sarcoma and hyperparathyroidism when GCT occurs in a typical locations in the skeletal system or in a long bone. The importance is that the management, prognosis, and therefore the final outcome differs radically.

Histology does not predict the extent of local aggression.[2] Histological grading of GCT of bone has not helped in predicting the outcome of treatment.[3]

Aneurysmal bone cyst (ABC) is a benign cystic lesion of bone that is composed of blood-filled spaces separated by connective tissue septa containing fibroblasts, osteoclast-type giant cells, and reactive woven bone.[2] ABC may be either primary or secondary. Primary ABCs are those which arise de novo. A secondary ABC develops in association with other neoplasms, most commonly GCT of bone, osteoblastoma, chondroblastoma, and fibrous dysplasia. ABC-like changes may also be found in sarcomas, especially osteosarcoma.[2]

When ABC occurs with a GCT of bone, the diagnosis is usually based only on histopathology. Cystic (secondary ABC) components are reported in 14% of GCTs.[4] It is difficult to identify this on a plain radiograph. If fluid-fluid levels are noted within the lesion on MRI, an ABC component may be suspected. There may be confusion regarding the diagnosis if tissue for biopsy is taken from this cystic region. However, the presence of ABC has no implications as far as the management or the outcome of treatment of GCT of bone is concerned.[5]
